# Predictive factors of selective mineralocorticoid receptor antagonist treatment in chronic central serous chorioretinopathy

**DOI:** 10.1038/s41598-020-73959-4

**Published:** 2020-10-06

**Authors:** Róbert Gergely, Illés Kovács, Zsuzsanna Récsán, Gábor László Sándor, Cecília Czakó, Zoltán Zsolt Nagy, Mónika Ecsedy

**Affiliations:** Department of Ophthalmology, Semmelweis University, 26 Üllői Street, 1085 Budapest, Hungary

**Keywords:** Biomarkers, Health care, Medical research

## Abstract

To compare the macular morphology of good and poor responders to eplerenone treatment in chronic central serous chorioretinopathy (CSCR) patients. Thirty eyes of 29 patients with chronic CSCR were treated with 50 mg/day oral eplerenone and followed up for 1 year. The integrity of outer retinal layers at baseline was assessed using optical coherence tomography. Patients who showed complete resolution of subretinal fluid at 1 year were assigned to the good responder group (Group 1), whilst those who showed moderate or no resolution were classified as poor responders (Group 2). Ellipsoid zone interruption, ELM interruption and hyperreflective foci in outer segment (OS) and outer nuclear layer (ON layer) was significantly more frequent in Group 2 than in Group 1 (p < 0.05 for all parameteres). Outer segment elongation was significantly more frequently seen in Group 1 than in Group 2 (p < 0.05) Multivariable regression analysis showed that intact ellipsoid zone at baseline is an independent predictor of good therapeutic response, with an odds ratio of 26.00 (95% CI 3.69–183.45; p = 0.001) after controlling for the effect of hyperreflective foci and ELM integrity. There is higher chance of the resolution of subretinal fluid after eplerenone treatment in CSCR patients with intact outer retinal layers at baseline. Baseline morphologic evaluation of the outer retinal layers on OCT scans can be useful in predicting the response to mineralocorticoid antagonist therapy in these patients.

## Introduction

In the last 5 years our understanding of the pathophysiology of CSCR has changed dramatically after discovering that aldosterone plays an important role in retinal homeostasis and that excessive occupancy of the mineralocorticoid receptor (MR) by glucocorticoids induces MR activation, causing dilatation and leakage of the choroid vessels^1–6^. Zhao et al. demonstrated that aldosterone controls the hydration of healthy retina by up-regulating the aquaporin 4 (AQP4) water channel in the retinal Müller cells through MR^6^. In another study Zhao et al. proved that aldosterone specifically affects the choroidal vascular bed, but not the retinal^7^. They showed that MR activation up-regulates the KCa2.3 channel that leads to hyperpolarization of endothelial cells and the underlying smooth muscle cells, thus inducing choroidal vasodilatation^7^. Based on these results, it was hypothesized that the MR-antagonist eplerenone could be a possible treatment in CSCR^7^. Bousquet et al. found significant choroidal thinning and visual improvement after 1 to 3 months of eplerenone treatment in 13 patients with chronic CSCR^8^. These results have been validated by numerous other studies^9–12^. We also showed in our previous prospective clinical study that 3 month eplerenone therapy can reverse the choroidal vasodilatation and subsequently induce reabsorption of subretinal fluid^13^.

Despite these promising results, we noticed that there are some patients who tend to react only modestly to treatment. This study was intended to assess if there are any factors which can differentiate the CSCR patients who would benefit promptly from eplerenone treatment.

## Methods

### Study design and patient recruitment

In this retrospective cohort study, we included 30 eyes of 29 patients with chronic CSCR, presented between June 2014 and September 2017 at the Department of Ophthalmology of Semmelweis University, Hungary, who were treated with 50 mg/day of oral eplerenone and followed for at least 1 year. Inclusion and exclusion criteria are summarized in Table 1. The patients who showed complete resolution of subretinal fluid at 1 year were assigned to the good responder group (Group 1), whilst those who showed moderate or no resolution were categorized as the poor responders (Group 2). CSCR patients were treated with oral eplenerone (Inspra, Pfizer) 25 mg/day for a week, followed by 50 mg/day for at least 3 months. Follow up visits were performed at 3-month intervals of 3, 6, 9, and 12 months. If any subretinal fluid was observed during spectral-domain optical coherence tomography (SD-OCT) examination after the 3-month eplerenone treatment, the therapy was continued or restarted up to the presence of subretinal fluid. The potential risks and benefits were thoroughly discussed with all patients and written informed consent was obtained before treatment. The study was conducted according to the Declaration of Helsinki, relevant national and local requirements and was approved by the Ethical Review Board for Human Research of the National Drug Agency (ETT TUKEB, study number 104/2014, clinicaltrial.gov identifier NCT02462499).

**Table 1:** Patient recruitment criteria.

**Inclusion criteria**
Visual symptoms and clinical diagnosis of CSCR for at least 3 months RPE changes typical of CSCR
Presence of SRF involving the fovea on OCT and the SRF did not dissolve during the 3-month period or did not decrease > 50 µm
**Exclusion criteria**
Under 18 years of age or limited capacity
Any evidence of choroidal neovascularization or choroidal hemangioma rescue PDT, anti-VEGF or laser treatment
History of other retinal abnormalities
Liver or kidney disease
Hyperkalemia (> 5.0 mmol/L)
High serum creatinine level (> 2 mg/dL in men, > 1.8 mg/dL in woman) or creatinine clearance < 50 mL/min
Concomitant therapy with drugs that increases the potassium level or are known to interact with eplerenone
Pregnancy or planning to conceive

### Baseline and follow-up examinations

Every patient included in the study had a physical examination and a routine blood test before treatment, with the former being repeated each month if the treatment was continued. We recorded the patient’s medical history, serum potassium level, and kidney function (estimated glomerular filtration rate). Previous ophthalmological treatment for CSCR (anti-VEGF, photodynamic therapy) was noted, if there was any. At baseline and at every follow-up, best-corrected visual acuity (BCVA) measurement with ETDRS charts at 4 m was performed on both eyes of each patient (Snellen acuity and ETDRS letter score were noted; 20/20 = 85). We also noted the duration of the disease and whether it was recurrent or not. The diagnosis of CSCR was based on slit lamp examination and optical coherence tomography (OCT) findings (localized and limited serous detachments of the neurosensory retina on the posterior pole, without any other findings). When needed for differential diagnosis, and to exclude choroidal neovascularization, we also performed fluorescein angiography and/or indocyanine green angiography. Macular morphology was examined with Spectral-Domain Optical Coherence Tomography (RTvue OCT ver.6, 11.0.12, Optovue Inc., Fremont, USA). Horizontal raster pattern scans centered on the fovea were used for the examination. The following morphological changes were noted: subfoveal and extrafoveal pigment epithelium detachments (PED), double layer signs (DLS), elongation of the photoreceptors’ outer segment, integrity of the external limiting membrane (ELM), integrity of the ellipsoid zone, hyperreflective foci in the outer segment (OS) and outer nuclear layer (ON layer), and subretinal deposits in the subretinal fluid. A layer was defined intact if there was no interruption in that layer on any of the OCT scans. Single or multifocal appearance was noted. Retinal thickness was measured automatically from the internal limiting membrane to Bruch’s membrane in all the 9 subfields of the macula. For data analysis we used the central subfield mean thickness of the Early Treatment Diabetic Retinopathy Study (ETDRS, the central 1.0 mm diameter of the macula). Data were recorded at baseline and then every 3 months for one year. Any adverse events that occurred were recorded at each visit.

### Outcome measures

The primary objective was to point out morphological differences seen on SD-OCT between the patients who responded well to eplerenone treatment and those who did not and to identify predictive factors of good therapeutic response.

The secondary objective was to assess the relationship between general risk factors (hypertension, hyperlipidemia), disease duration and reoccurrence with regards to treatment response.

### Statistical analysis

Statistical analysis was performed with Statistica software (version 13.2, Statsoft Inc., Tulsa, OK, USA). The independent samples student’s t-test, ANOVA and chi-squared test were applied for group comparisons. The paired samples t-test was used for statistical comparisons of repeated measurements. The effect of predisposing factors on treatment response was assessed with multivariable regression analysis using binomial logit models to estimate odds of good response after controlling for the effect of other risk factors. Covariates which were evaluated to be potential confounders were included where the parameters showed significant difference in group comparisons. In all statistical analyses, a p-value of less than 0.05 was considered to be statistically significant.

### Statement of ethics

The study was conducted according to the Declaration of Helsinki, relevant national and local requirements, and was approved by the Ethical Review Board for Human Research of the National Drug Agency (ETT TUKEB, study number 104/2014, clinicaltrial.gov identifier NCT02462499).

## Results

Thirty eyes of 29 patients were included in the study. In the whole cohort, central retinal thickness decreased significantly (p = 0.0001). The central subfield mean thickness decreased in both groups, but the change was more significant in good responder patients (Group 1). We found complete resolution of subretinal fluid in 15 eyes (51.72%) of 14 patients (Group 1), and 15 eyes of 15 patients showed no (less then 50 µm subretinal fluid absorptionn) or just moderate resolution (more than 50 µm, but not full subretinal fluid absorption) (Group 2).

There was no significant difference in baseline central subfield macular thickness, baseline visual acuity, disease duration, recurrence, treatment duration and the presence of general risk factors (hypertension, hyperlipidemia) between the two groups.

Baseline characteristics, demographic data, patient history, disease duration and treatment duration in the two groups are summarized in Table 2.

**Table 2:** Baseline, disease, and treatment characteristics of the two groups.

	Good responders (Group 1, n = 15)	Poor responders (Group 2, n = 15)	p
Age/years (mean ± SD)	55.06 ± 12.89	53.73 ± 9.62	0.75
Male (n)	11	10	0.69
Female (n)	4	5	0.69
Hypertension (n)	7	6	0.71
Diabetes (n)	3	2	0.62
Disease duration/months (mean, min–max)	54.73 (4–80)	52.2 (4–166)	0.35
Recurrent form (n)	10	10	0.99
Baseline visual acuity ETDRS letters (mean ± SD)	76 ± 14.04	69.26 ± 9.39	0.13
Baseline CSMT (µm)	362.6 ± 71.60	355.67 ± 106.51	0.85
Treatment duration (months) (mean, min–max)	4.2 (3–12)	5.53 (3–12)	0.19

Independent samples student’s t-test and chi-squared test were applied for group comparisons.

Patients in Group 1 gained on average 5.0 letters at 12 months compared to baseline, while Group 2 showed a decrease in visual acuity of 5.9 letters on average. Visual acuity change was marginally significantly higher in Group 1 than in Group 2 (5 patients and 1 respectively), when comparing 10 letter change in visual acuity (p = 0.06). Full visual acuity recovery to 20/20 BCVA was significantly more often seen in Group 1 than in Group 2 (12 patients versus 1 patient, p = 0.0001).

**Figure 1:**
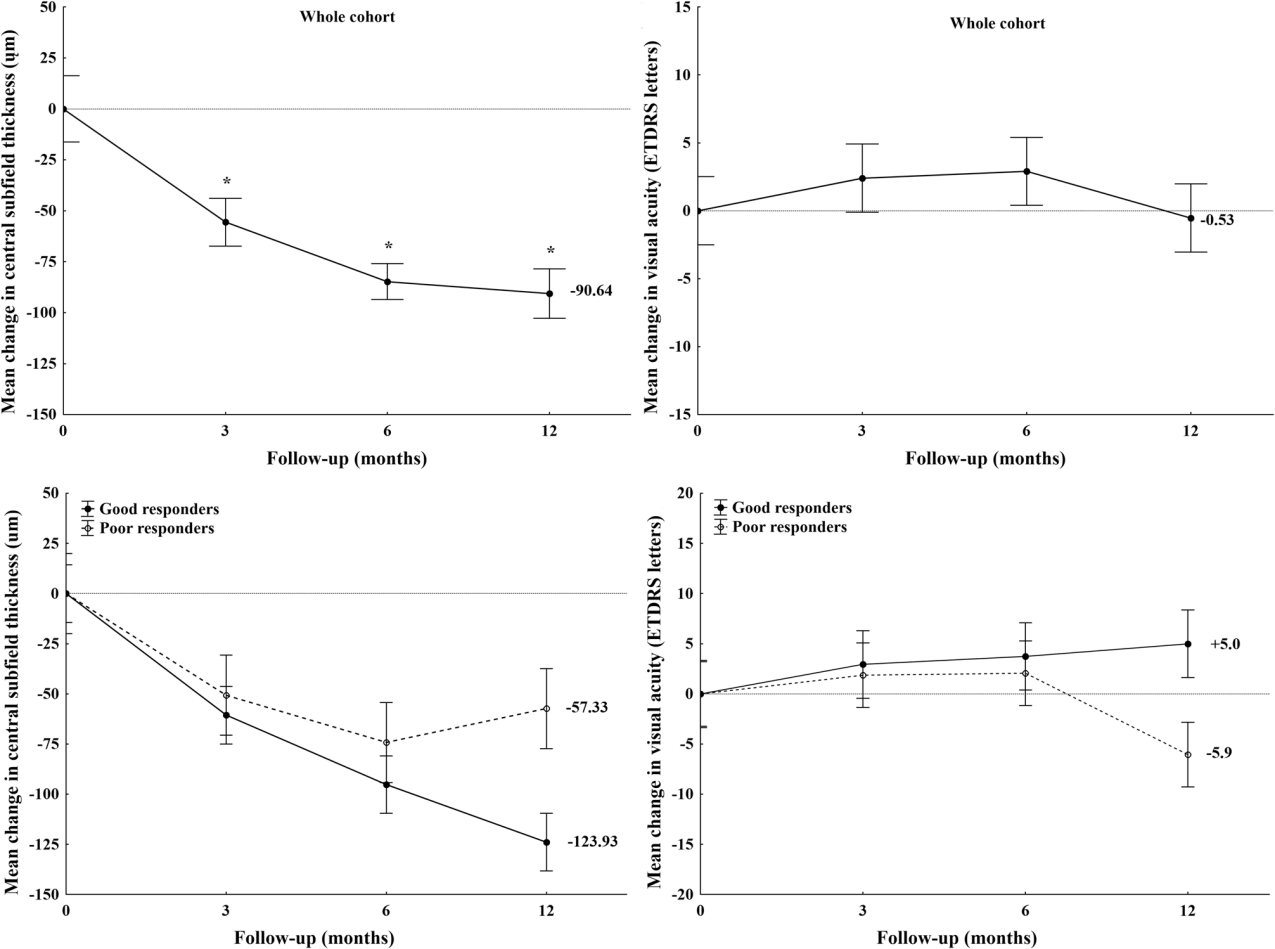
Visual acuity and central subfield thickness (CSMT) changes in the whole cohort (top images), Visual acuity and central subfield thickness (CSMT) changes in the two study groups (bottom images).

Changes in central subfield mean thickness (CSMT) and visual acuity are summarized in Table 3 and Fig. 1A–D.

**Table 3:** Visual acuity and central subfield thickness (CSMT) during the follow-up.

	Baseline	Month 3	Month 6	Month 12
**Whole cohort**				
Visual acuity (ETDRS letter)	72.63 ± 12.22	75.03 ± 12.83	75.53 ± 13.05	72.10 ± 16.44
CSMT (µm)	359.13 ± 89.24	303.50 ± 63.96	274.37 ± 47.67	268.50 ± 64.57*
**Good responders**				
Visual acuity (ETDRS letter)	76.00 ± 14.04	78.93 ± 13.39	79.73 ± 13.62	81.00 ± 10.72*
CSMT (µm)	362.60 ± 71.60	302.00 ± 60.85	267.33 ± 39.11	238.67 ± 44.44*
**Poor responders**				
Visual acuity (ETDRS letter)	69.27 ± 9.39	71.13 ± 11.36	71.33 ± 11.37	63.20 ± 16.58^†^
CSMT (µm)	355.67 ± 106.51	305.00 ± 69.06	281.40 ± 55.41	298.33 ± 68.87^†^

*p < 0.01 compared to baseline using repeated measures analysis of variance test.

^†^p < 0.01 compared to good responders using the independent samples student’s t-test.

**Figure 2:**
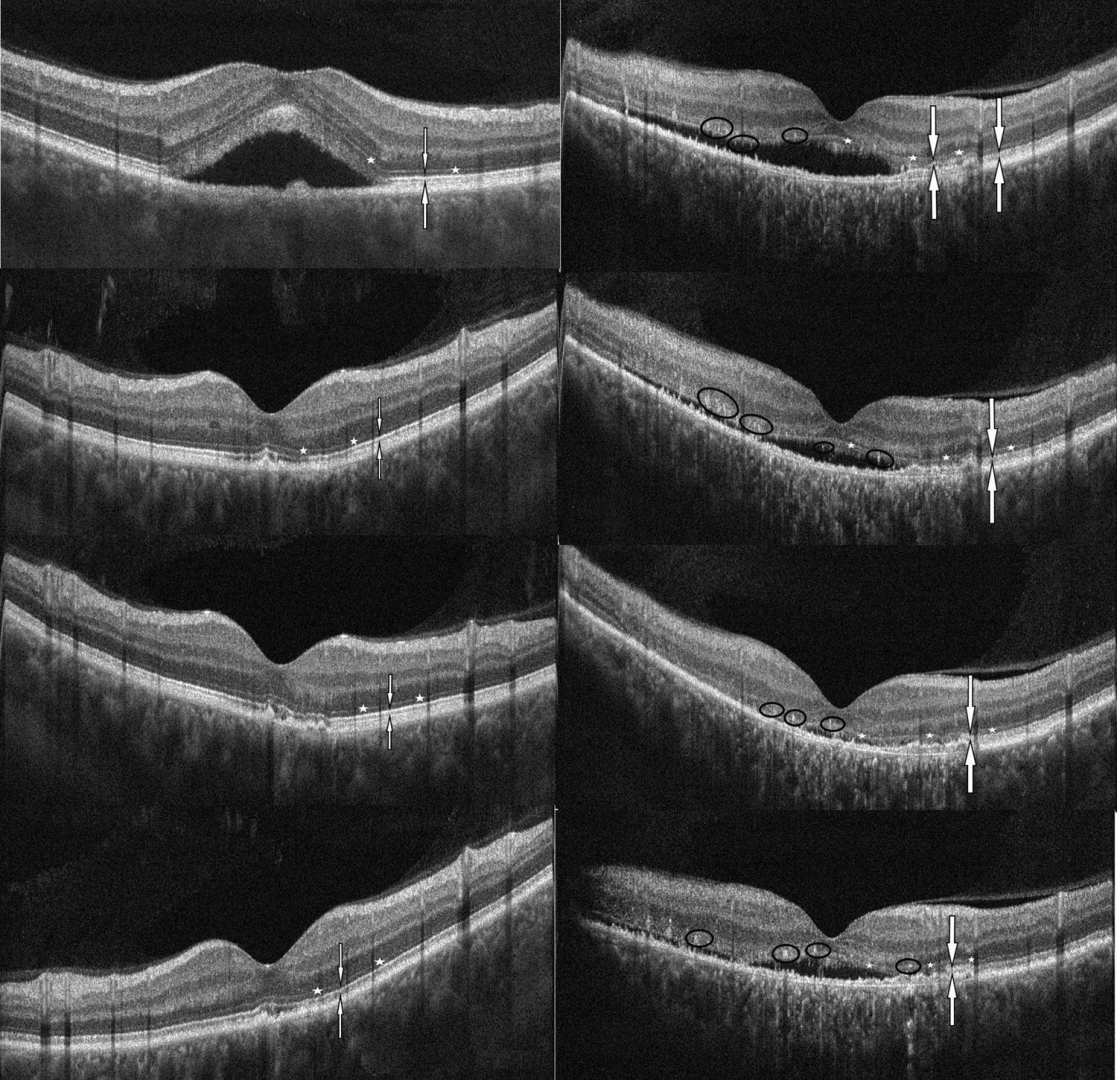
OCT images of good responder to eplerenone at 0, 3, 6 and 12 months: baseline SRF was absorbed after 3 months therapy and did not reoccure:ellipsoid is intact (line between white arrows), ELM is intact (little white stars) (left images from top to bottom). V OCT images of poor responder to eplerenone at 0, 3, 6, and 12 months: ellipsoid zone is interrupted (line between white arrows), hyperreflective foci in the OS and ON layer, ELM is slightly damaged (little white stars) (right images from top to bottom).

Ellipsoid zone and ELM integrity and hyperreflective foci in the OS and ON layer (also known as intra and subretinal precipitates) were found to be significant factors in differentiating mineralocorticoid receptor antagonist treatment response. In patients who showed complete resolution of subretinal fluid at the 1-year follow-up, a continuous ellipsoid zone, an intact ELM and an elongated OS layer was observed significantly more frequently at baseline, whilst hyperreflective foci in the OS and ON layer was observed to be significantly less frequent than that recorded in the poor responder group (Table 4, Fig. 2).

**Table 4:** Baseline OCT characteristics of the two study groups.

	Good responders (n = 15)	Poor responders (n = 15)	p
PED	13	14	0.54
Double layer sign	10	12	0.41
OS elongation	13	8	0.04
Hyperreflective foci in OS and ON layer	9	15	0.006
Subretinal deposits	2	2	0.99
Ellipsoid integrity	12	2	< 0.001
ELM integrity	12	6	0.02

*PED* pigment epithelial detachment, *OS* outer segment, *ON layer* outer nuclear layer, *ELM* external limiting membrane.

p value calculated using chi-squared test.

There was no statistically significant difference between the groups regarding the double layer sign (Table 4).

Finally, multivariable binomial logistic regression analysis was performed to determine the most important significant predictor of good anatomical response to eplerenone treatment. According to the final model baseline intact ellipsoid layer was found to be a significant predictor of total fluid resolution (odds ratio: 26.00, 95% CI 3.69–183.45; p = 0.001) independently of hyperreflective foci and ELM integrity (r^2^: 0.43; p < 0.001).

### Safety analysis

There were no serious events that occurred as a result of eplerenone treatment. Mild adverse events were seen in 20.68% of the cases (dry mouth, dizziness, back pain, and sleepiness for a few hours after taking the pills). The treatment with eplerenone was generally well tolerated and did not lead to elevated potassium levels or low creatinine clearance.

## Discussion

In the last five years many papers showed that eplerenone is a safe and effective treatment option in chronic CSCR patients, mainly through reversing the choroidal vasodilatation that—results in resolution of subretinal fluid^1,7–13^.

In this study, we have chosen fluid resolution as the biomarker of good response to treatment instead of the change in visual acuity, because neuroretinal re-attachment is not always accompanied by visual acuity improvement. Besides the presence of subretinal fluid several other factors are influencing the visual prognosis, such as the disruption of ellipsoid zone, disruption of the ELM, thinning of the ON layer and photoreceptor outer segment, hyperreflective foci in the retinal layers and the severity of RPE atrophy^1,15^.

Recent studies reported different predictors of treatment response in this new therapy. In our previous prospective study we found that in eplerenone treated CSCR patients baseline choroidal thickness was a positive predictive factor for subretinal fluid decrease, results which were recently supported by Bousquet et al. who found that a thick choroid at baseline is associated with good treatment response^13,14^. In our previous pilot study we found that outer segment elongation and baseline visual acuity also proved to be a significant predictor of good visual acuity^15^. Regarding neuroretinal morphological changes Cakir et al. reported that CSCR patients with intact RPE and intact ellipsoid zone had better BCVA after eplerenone treatment^16^. Matsumoto et al. showed that the ON layer thickness positively correlates with the visual acuity in resolved CSCR and the discontinuity in the ellipsoid zone is associated with worse visual acuity^17^. A recent study reviewed clinical and multimodal imaging data of 133 patients with CSCR and found that outer retinal disruption on OCT is significantly associated with poorer visual acuity^18^.

Sacconi et al. reported possible biomarkers on ICG-angiography and OCT-angiography which could predict the response to eplerenone treatment in CSCR patients^19^. They found that the absence of choroidal neovascularization (CNV) on OCT-angiography and the presence of hot spot on ICG-angiography were associated with a good response to treatment, with 58% of the patients (17 out of 29) showing a complete resolution of the subretinal fluid at the end of the 13 week treatment^19^. Interestingly patients with CNV and hotspot also showed a moderate response to eplerenone therapy^19^.

In our study population ellipsoid zone integrity at baseline was found to be a crucial and independent predictor of good anatomical treatment response as defined by complete and stable resolution of subretinal fluid. At the same time, hyperreflective foci in the OS and ON layer showed a significant negative correlation with subretinal fluid resolution.

Within the outer retina, a hyperreflective band—previously thought to represent the photoreceptor inner/outer segment (IS/OS) junction, more commonly referred now as the ellipsoid zone—can be observed on SD-OCT. Spaide et al. showed that this layer most likely represents the photoreceptors inner segments ellipsoid part (closer to the outer segment) which is rich in mitochondria, organelle reliable of cells energy and metabolism^20^. The integrity of this layer has been found to be of high clinical importance in the diagnostic and prognostic evaluation of various surgical and medical retinal diseases. The absence or disruption of this layer has been found to be associated with lower visual acuity and poor visual prognosis^21–23^. An intact ellipsoid layer was identified as a significant biomarker for macular hole, epiretinal membrane and retinal detachment surgery, as well as eyes with wet age-related macular degeneration, retinal vein occlusion or diabetic macular edema^21^. Damage of ellipsoid zone integrity was described in the natural course of CSCR, which also correlates with the macular function evaluated by microperimetry^22,23^. In several SD-OCT studies, the ellipsoid zone was not visible in eyes with acute serous detachment, though it did became visible after its resolution^22,24,25^. Koo et al. suggested that the membranous stack of the photoreceptor segment is no longer perpendicular to the incoming OCT beam in acute neurosensory retinal detachment, as the highly back-reflecting signal at the IS/OS seems to be absent in the area of detachment^26^. Accordingly, even if an eye with active CSCR does not show a distinct ellipsoid zone, good visual acuity is often obtained when the subretinal fluid is reabsorbed^26,27^. Ellipsoid recovering capacity in CSCR patients shows us that photoreceptors can be preserved for quite long time despite their long-lasting separation from retinal pigment epithelium. In an experimental animal model of retinal detachment a significant decrease in the number of photoreceptors nuclei in the ON layer was observed at 1 month, followed by outer plexiform layer (OP layer) degeneration within 50 days due to necrosis of cell processes and synaptic terminals^28^. According to Piccolino et al.’s study, this is not the case in CSCR eyes where SD-OCT does not show atrophy of the OPL in detachments lasting several months and that a severe loss of the photoreceptor layer only being observed in patients with symptoms lasting more than 1 year^25^. In our study, baseline ellipsoid layer damage shows a significant positive correlation with poor anatomical response to MR-antagonist treatment. We also found that baseline outer segment elongation shows a significant positive correlation with subretinal fluid resolution after eplerenone treatment. In this context outer segment elongation can be considered as a predictor of good anatomical response to eplerenone treatment. OS elongation—which was earlier described as the only sign of neuroretinal changes in acute CSCR patients seems to be reversible when photoreceptors are injured but not degenerated. Our observations about the loss of ellipsoid zone integrity and the attenuation of the elongated OS layer in the poor responder group supports the hypothesis that these two layers morphological changes are directly proportional with the progressive loss of photoreceptors function resulting not only in bad visual prognosis, but also in poor response to MR-antagonist treatment.

Hyperreflective foci in the OS and ON layer, as observed in all patients in the poor responder group, were the other significant biomarker predicting poor treatment response in our study population. These abnormal features of the OS and ON layer were earlier reported on the time-domain Stratus OCT images as a granulated profile of the outer border of neurosensory retina, mostly attenuated to the chronic or recurrent form of the disease^25^. Later in SD-OCT studies these hyperreflective foci in the OS and ON layer were identified in as many as 65% of chronic CSCR eyes in the area of serous retinal detachment and it co-localized with hyperautofluorescent areas on FAF images^24,29–32^. The nature of these dots vary depending on their location^32^. In the subretinal space these dots are thought to be macrophages and microglia activated by the photoreceptor outer segments shedding^31^. Concentration of protein-like compounds, fibrin or lipids could also be identified as dots^29^. Recently the observation of these hyperreflective foci in active and resolved CSCR eyes with adaptive optics scanning light ophthalmoscopy supported the hypothesis that they are cellular in nature, corresponding to activated microglia cells or macrophages^33,34^. Their presence were found to be correlated not only to disease duration, but also to poorer final visual acuity^17,35^.

Interestingly, despite the fact that both ellipsoid zone and ELM deterioration classically is related to longer disease duration, no significant difference was found in disease duration or recurrence between the good and poor responder groups. One explanation for these results could be that in recurrent and chronic CSCR the duration of neurosensory detachment is often doubtful. The other important factor is that until now no consensus existed over the duration threshold that differentiates acute and chronic CSCR. In most published reports, it was arbitrarily set as being between 3 and 6 months and was used as a limit in interventional studies to determine the appropriate timing for treatment, in order to avoid self-resolution cases. As a result of which we consider that a consensual definition of the various clinical subtypes of CSCR is needed as their exact limits are critical in the determination of clinical trial design. We are of the opinion that morphologic assessment might better differentiate acute from chronic forms given that the duration of the disease seems to be an interval rather than an exact time following the appearance of the disease. These data suggests that the disease duration does not affect directly the response to eplerenone treatment.

Our patients baseline visual acuity was quite good despite the chronic presence of subretinal fluid, so for this reason the change in visual acuity was smaller than one could expect from an effective therapy. On the other hand the analysis of the two groups showed that if subretinal fluid resolution is complete after eplerenone therapy a full visual acuity recovery to 20/20 BCVA is significantly more often seen in patients who had a baseline intact ellipsoid zone, an elongated OS layer and no hyperreflective foci in OS and ON layer. These results probably can help us in the future to form a more precise opinion to our patients about their chances of good anatomical and visual recovery right at the beginning of MR-antagonist treatment. These patients are alarmed by any irreversible vision deterioration which further can enhance the disease progression; a fair and positive reassurance at the beginning of therapy might be beneficial regarding the outcome, knowing that the elimination of any stress factor is mandatory in the healing process of CSCR. On the other hand, for a complete transparency with the patient, one should take into consideration the conclusions of other papers too, which suggests that eplerenone might not be such an effective treatment option for CSCR^36^.

To our knowledge we provide new data regarding the correlation between changes in the ellipsoid zone and outer segments and the anatomical response to eplerenone treatment in CSCR patients.

Our study’s limitations are the small sample size, the retrospective design and the absence of a placebo-treated group. Nevertheless our cohort consisted of strictly selected cases and was followed-up for a relatively long period.

## Conclusions

Our results suggest that in chronic or recurrent CSCR a morphologic evaluation of the macula may provide useful information not only about the present state of photoreceptor deterioration and potential for visual recovery, but also about the probability of subretinal fluid resolution. Next to choroidal thickness measurement the ellipsoid zone seems to be a useful biomarker for selecting the CSCR patients who could benefit the most from mineralocorticoid antagonist therapy.
